# Sensitive Detection of Genotoxic Substances in Complex Food Matrices by Multiparametric High-Content Analysis

**DOI:** 10.3390/molecules29143257

**Published:** 2024-07-10

**Authors:** Pengxia Gao, Zhi Li, Mengqiang Gong, Bo Ma, Hua Xu, Lili Wang, Jianwei Xie

**Affiliations:** 1 Laboratory of Toxicant Analysis, Academy of Military Medical Sciences, Beijing 100850, China; 2 School of Chemistry and Pharmaceutical Engineering, Hebei University of Science and Technology, Shijiazhuang 050018, China

**Keywords:** genotoxicity, high-content analysis, γ-H2AX, p-H3, RAD51, complex matrices

## Abstract

Genotoxic substances widely exist in the environment and the food supply, posing serious health risks due to their potential to induce DNA damage and cancer. Traditional genotoxicity assays, while valuable, are limited by insufficient sensitivity, specificity, and efficiency, particularly when applied to complex food matrices. This study introduces a multiparametric high-content analysis (HCA) for the detection of genotoxic substances in complex food matrices. The developed assay measures three genotoxic biomarkers, including γ-H2AX, p-H3, and RAD51, which enhances the sensitivity and accuracy of genotoxicity screening. Moreover, the assay effectively distinguishes genotoxic compounds with different modes of action, which not only offers a more comprehensive assessment of DNA damage and the cellular response to genotoxic stress but also provides new insights into the exploration of genotoxicity mechanisms. Notably, the five tested food matrices, including coffee, tea, pak choi, spinach, and tomato, were found not to interfere with the detection of these biomarkers under proper dilution ratios, validating the robustness and reliability of the assay for the screening of genotoxic compounds in the food industry. The integration of multiple biomarkers with HCA provides an efficient method for detecting and assessing genotoxic substances in the food supply, with potential applications in toxicology research and food safety.

## 1. Introduction

Genotoxic substances are ubiquitous in daily life and the environment, posing a significant threat to human health due to their potential to cause DNA damage, gene mutations, and carcinogenesis. The public’s concern over genotoxicity is growing, particularly in relation to the presence of these substances in the food supply and their high association with cancers. Such substances can originate from food additives, such as nitrites, or be introduced as contaminants during the production, processing, and storage of food, with examples including aflatoxins, acrylamide, polycyclic aromatic hydrocarbons (PAHs), and heterocyclic amines (HAs). Traditional in vitro assays for genotoxicity assessment, including the Ames assay, comet assay, mouse lymphoma assay, micronucleus assay, and chromosome aberration assay, have been widely employed. However, these methods are often laborious and have limited throughput, necessitating the development of more efficient alternatives.

Advances in technology have led to the development of high-throughput assays that offer improved efficiency. For instance, an In-Cell Western technique has been adapted to detect a panel of cellular biomarkers indicative of genotoxicity, including phosphorylated H2AX (γ-H2AX) and Ser-10 phosphorylated histone H3 (p-H3) [1,2,3,4]. Despite these advancements, challenges remain regarding the sensitivity and specificity of these assays, particularly when applied to complex food matrices. Therefore, it is urgent to establish a sensitive and convenient method to detect genotoxic substances in complex matrices.

The detection of DNA double-strand breaks (DSBs) is a critical aspect of genotoxicity assessment. DSBs trigger the rapid phosphorylation of serine 139 on histone H2AX, resulting in the formation of γ-H2AX. The quantity of γ-H2AX foci correlates with the extent of DSBs, making it a reliable biomarker for genotoxicity evaluation [5,6]. Additionally, aneuploid inducers lead to delayed mitosis and significant formation of p-H3, a biomarker for cell mitosis, making it a potential indicator of aneuploidy. Many studies have shown that the simultaneous detection of γ-H2AX and p-H3 can effectively distinguish genotoxic compounds with different modes of action [1,7]. When cells are damaged by genotoxic agents, such as mitomycin C, ultraviolet rays, or ionizing radiation, the RAD51 protein will aggregate at DNA break sites for the repair process through homologous recombination [8].

As a biomarker of DNA damage, γ-H2AX promotes the recruitment of repair and cell cycle proteins to the damage site during DNA damage repair (DDR). The γ-H2AX assay is a rapid and accurate assay for detecting DNA damage [9]. Several studies have reported on the intracellular detection of γ-H2AX by different techniques, such as flow cytometry [10], whole-cell enzyme-linked immunosorbent assay [11], and bidirectional gel electrophoresis [12]. p-H3, an aneuploidy biomarker, is closely related to the cell cycle and gene transcription regulation. Recent studies have shown that a combination of the two biomarkers can distinguish compounds causing chromosome breakage from aneuploid induced by antibody-based methods [13]. Qu et al. used stable isotope dilution-liquid chromatography–tandem mass spectrometry for the dynamic detection of genotoxic compounds and found that the simultaneous analysis of γ-H2AX and p-H3 could distinguish between chromosome breakage-causing compounds and aneuploidy compounds [14]. Homologous recombination (HR) is the main repair mechanism for DNA double-strand damage and is important for maintaining genomic integrity in mammalian cells [15]. RAD51 is a central molecule of HR, which participates in a complex signaling network for DNA damage sensing and cell cycle regulation [16].

High-content analysis (HCA) is a powerful tool that enables the quantitative assessment of cellular toxicity at a subcellular level with microscopic resolution. It has demonstrated high sensitivity and specificity and is capable of measuring multiple cellular events within individual cells. HCA plays an important role in toxicology research, cell biology, and many other fields and is recognized as a valuable tool for toxicity testing and chemical risk assessment [17]. In this study, a multiparametric high-content analysis measuring γ-H2AX, p-H3, and RAD51 was developed to sensitively detect genotoxic substances in various complex food matrices. This novel assay not only enhances the ability to distinguish genotoxic compounds with different modes of action, but also provides new insights into the exploration and evaluation of genotoxicity mechanisms. The integration of these biomarkers into a single, high-throughput assay offers a more comprehensive and efficient means of assessing potential health risks associated with genotoxic substances in the food supply.

## 2. Results and Discussion

### 2.1. Establishment of a Multiparametric High-Content Analysis for Genotoxic Substances Detection

**Figure 1:**
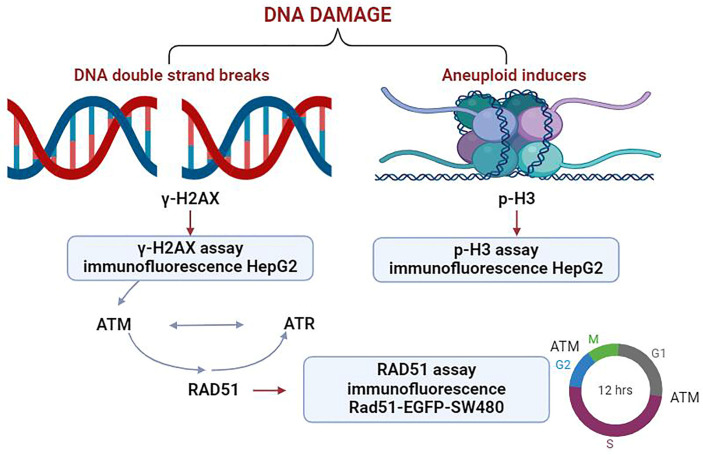
The multiparametric high-content analysis strategy for genotoxic substances detection developed in this study.

This study utilized a panel of biomarkers, including γ-H2AX, p-H3, and RAD51, to develop an integrated analysis strategy (Figure 1). Spectra of genotoxic substances with distinct mechanisms of action were selected as model compounds. These included DNA adduct-forming agents, spindle poisons, kinase inhibitors, and nucleoside analogs, which collectively provide a robust framework for the assessment of genotoxicity in complex biological contexts. The results showed that the combination of the three biomarkers could detect all classes of genotoxic substances. The sensitivity of this assay was 100% (12/12).

### 2.2. In Vitro Assessment of γ-H2AX as a DNA Damage Marker

**Figure 2:**
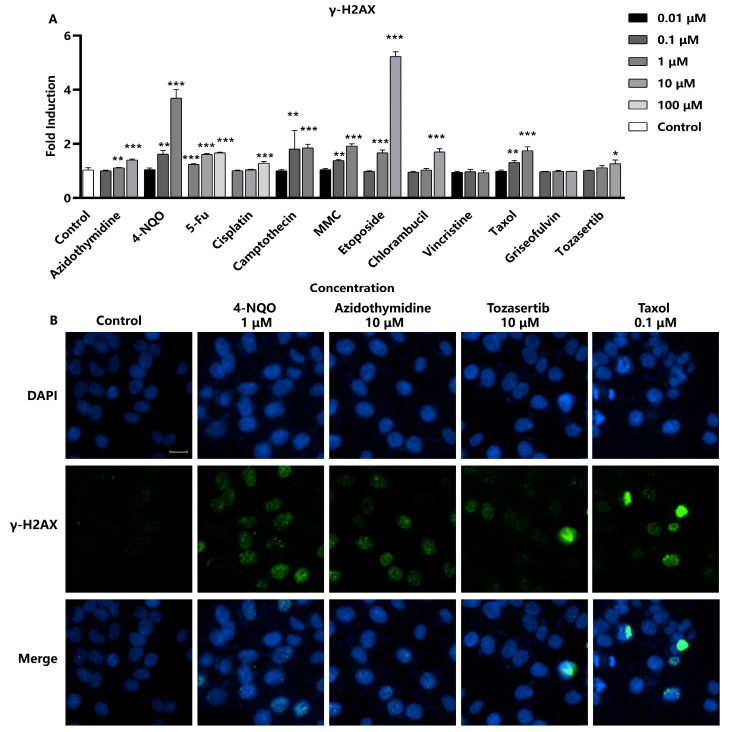
In vitro assessment of γ-H2AX as a DNA damage marker. Hep G2 cells were seeded into 96-well black-bottomed plates at a density of 1.5 × 10^4^ cells/well and exposed to various compounds. (**A**) The fluorescence intensity of γ-H2AX in the nuclei was measured and calculated as fold induction compared with control group. Statistical significance was determined using one-way ANOVA (* *p* < 0.05, ** *p* < 0.01, *** *p* < 0.001 vs. control). (**B**) The representative images were captured using a high-content cell analysis system. Scale bar: 20 μm.

To assess the efficacy of γ-H2AX as a marker for DNA damage, Hep G2 cells were exposed to 12 different genotoxic substances for 24 h. The induction of γ-H2AX in response to compound treatment was monitored, and quantitative analyses were performed to establish relationships between compound exposure and γ-H2AX levels (Figure 2A). Several studies have shown that certain compounds, such as Camptothecin and Etoposide, interact with DNA to form complexes, leading to double-strand breaks. Nitrogen mustard phenylbutyrate, Cisplatin, Mitomycin C (MMC), and 4-Nitroquinoline N-oxide (4-NQO) are bioalkylating agents, which covalently bind to DNA and lead to DNA breaks. The nucleoside analogs 5-fluorouracil (5-Fu) and azidothymidine (AZT), as well as all eight chromosome breakage-causing genotoxicity compounds, induce the production of γ-H2AX [18,19,20]. A concentration-dependent increase in γ-H2AX formation was observed, which is consistent with literature reports. In addition, two aneuploid-inducing compounds, Taxol and Tozasertib, were also observed to significantly increase γ-H2AX fluorescence intensity. However, the morphology of γ-H2AX foci induced by these aneuploid compounds was distinct from the morphology caused by chromosome breakage agents, and γ-H2AX expression was noted only at the highest concentration. As shown in Figure 2B, the entire nuclei of the aneuploid genotoxic substance Taxol-exposed cells exhibited a strong γ-H2AX fluorescent signal, which differs from γ-H2AX foci caused by other chromosome-breaking compounds. This observation may suggest a potential link between high-level γ-H2AX and apoptosis [21,22]. In conclusion, the sensitivity of γ-H2AX formation as a biomarker for genotoxic substances was 100% (8/8) for the chromosome breakage-causing compounds after a 24 h cell exposure period. When considering all 12 genotoxic substances, including four aneuploid agents, the specificity of the γ-H2AX assay was calculated to be 83.3% (10/12), demonstrating its utility as a robust and specific biomarker for genotoxicity assessment.

### 2.3. Assessment of p-H3 as a Marker for DNA Damage Induced by Aneuploid Genotoxic Compounds

**Figure 3:**
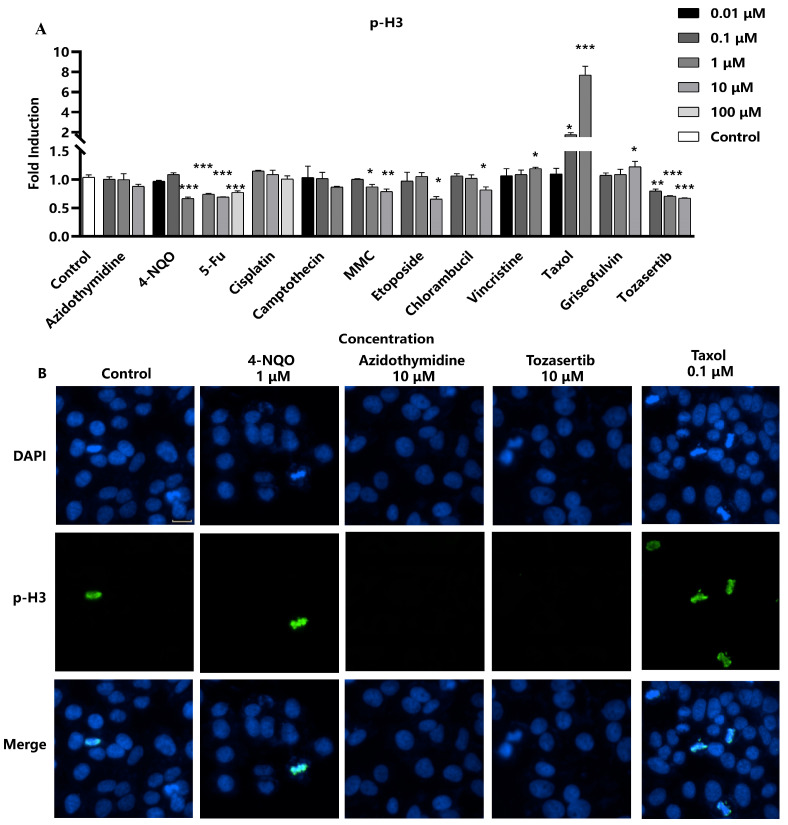
Assessment of p-H3 as a marker for DNA damage induced by aneuploid genotoxic compounds. Hep G2 cells were seeded into 96-well black-bottomed plates at a density of 1.5 × 10^4^ cells/well and exposed to various compounds. (**A**) The fluorescence intensity of p-H3 in the nuclei was measured and calculated as fold induction compared with the control group. Statistical significance was determined using one-way ANOVA (* *p* < 0.05, ** *p* < 0.01, *** *p* < 0.001 vs. control). (**B**) The representative images were captured using a high-content cell analysis system. Scale bar: 20 μm.

Next, the efficacy of p-H3 as a marker for aneuploidy was evaluated, particularly focusing on aneuploid genotoxic compounds with distinct mechanisms of action. Four aneuploid genotoxic compounds were selected, including Taxol, griseofulvin, and vincristine, which are all recognized as spindle poisons due to their interaction with microtubule proteins, thereby inhibiting spindle formation, as well as Tozasertib, an inhibitor of aurora kinase, which leads to mitotic abnormalities [15]. The results showed that four aneuploid compounds significantly impacted p-H3 levels. Specifically, the three spindle poisons exhibited a marked dose-dependent increase in p-H3 fluorescence intensity. Conversely, the kinase inhibitor Tozasertib demonstrated a dose-dependent decrease in p-H3, in accordance with the literature data (Figure 3) [15]. However, there was no evident relationship between concentration and p-H3 levels observed in the remaining compounds. Consequently, the sensitivity of the assay in detecting p-H3 formation induced by these aneuploid genotoxic compounds was 100% (4/4), underscoring its potential utility as a reliable marker for assessing DNA damage associated with aneuploidy.

### 2.4. Detection of RAD51 as a Marker for DNA Repair in Response to Genotoxic Stress

**Figure 4:**
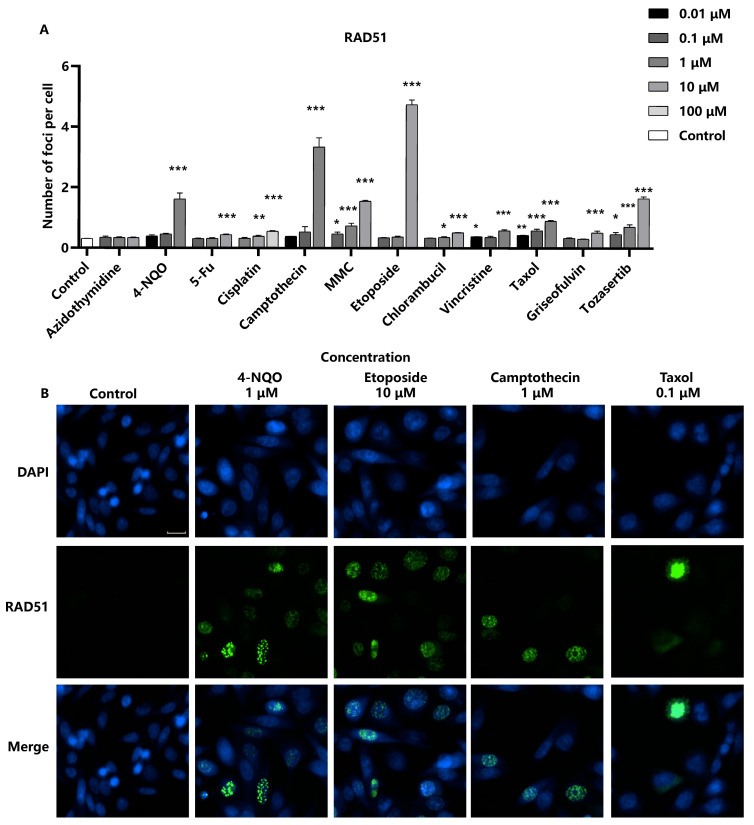
Detection of RAD51 as a marker for DNA repair in response to genotoxic stress. RAD51-EGFP-SW480 cells were seeded into 96-well black-bottomed plates at a density of 2.0 × 10^4^ cells/well and exposed to various compounds. (**A**) The average number of RAD51 foci in the nuclei per cell was measured. Statistical significance was determined using one-way ANOVA (* *p* < 0.05, ** *p* < 0.01, *** *p* < 0.001 vs. control). (**B**) The representative images were captured using a high-content cell analysis system. Scale bar: 20 μm.

When cells are damaged by genotoxic agents, ultraviolet light, or ionizing radiation, the RAD51 protein aggregates to DNA break sites to repair homologous sequences [23]. In our analysis, chromosome breakage-inducing compounds led to a significant increase in the number of RAD51 foci in the nuclei in a concentration-dependent manner, indicating the activation of the homologous recombination repair pathway in response to DNA damage. However, aneuploid compounds were found to increase RAD51 foci only at high concentrations (Figure 4). This observation may suggest a differential effect on the homologous recombination repair pathway compared to chromosome breakage-inducing agents. The sensitivity of detecting RAD51 foci formation induced by the panel of genotoxic substances was 91.6% (11/12). Moreover, the limit of detection (LOD) for genotoxic substances could be reduced and improved by integrated detection of γ-H2AX and RAD51. For compounds such as Cisplatin and Chlorambucil, the LOD of RAD51 was 10 times lower than that of γ-H2AX. It is noteworthy that DNA damage can activate various repair pathways, and homologous recombination is only one of them. Our study focuses on the homologous recombination pathway and the role of RAD51; however, homologous recombination is not the sole mechanism at play. Other pathways or proteins may be tested in future investigations.

### 2.5. Distinguishing Genotoxic Compounds with Different Modes of Action through Multiple Biomarkers

To further verify that compounds with different modes of action have different impacts on γ-H2AX and p-H3, various compounds were selected for comparison. To better interpret the effects of compounds on γ-H2AX and p-H3 with different modes of action, the results are summarized in Table 1, with yellow indicating an increase and blue indicating a decrease. Based on the combined analysis of γ-H2AX and p-H3, chromosome breakage-causing compounds can be effectively distinguished from aneuploidy compounds.

DNA double strand breaks and blocked or collapsed DNA replication forks are potentially genotoxic lesions that can result in deletion, aneuploidy, or cell death. Homologous recombination (HR) is an essential process employed during the repair of these forms of damage [24]. RAD51 is a central HR molecule that participates in a complex signaling pathway for DNA damage sensing and cell cycle regulation. Our study reveals a significant correlation between the levels of RAD51 and γ-H2AX. The expression trends of RAD51 were found to mirror those of γ-H2AX, underscoring their concurrent involvement in the DNA damage response. For example, all eight chromosome disruption compounds caused a dose-dependent increase in both γ-H2AX and RAD51. Notably, for the compounds Cisplatin and Chlorambucil, the lowest detectable concentrations of RAD51 were lower than those required for the detection of γ-H2AX. This observation suggests that the inclusion of RAD51 in conjunction with γ-H2AX may enhance the sensitivity and accuracy of genotoxicity assays. By integrating the three biomarkers into our analysis, we aimed to refine the detection methods, offering a more comprehensive assessment of DNA damage and the cellular response to genotoxic stress. This multi-biomarker approach holds promise for improved sensitivity, specificity, and accuracy in identifying compounds that pose a genotoxic risk, thereby contributing to the precision and reliability of toxicological evaluations.

### 2.6. Impact of Food Matrices on the Detection System

Assessing the impact of food consumption on the biological effects of genotoxicants is relevant for understanding the practical implications of our findings. Evaluating whether the food matrix can influence the detection system is also crucial for the reliability of the method in various dietary contexts. To determine if the integrated assay can be effectively utilized for the screening and detection of genotoxic substances in food, the potential influence of five distinct food matrices on the detection system was assessed, including coffee, tea, pak choi, spinach, and tomato.

**Figure 5:**
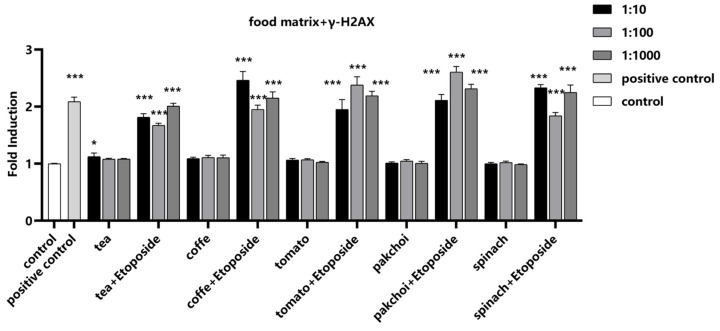
Impact of food matrices on the detection of γ-H2AX. Food matrices, including coffee, tea, pak choi, spinach, and tomato, were serially diluted to 1:10, 1:100, and 1:1000 with serum-free medium. The diluted matrices were spiked with positive control etoposide (1 μM). The fluorescence intensity of γ-H2AX was measured and calculated as fold induction compared with control group. Statistical significance was determined using one-way ANOVA (* *p* < 0.05, *** *p* < 0.001 vs. control).

To investigate the impact of food matrices on the detection of γ-H2AX, the well-known inducer of chromosomal breakage, Etoposide, was selected as a positive control. Etoposide was spiked into the food matrices at a final concentration of 1 μM. Notably, the food matrices themselves did not induce γ-H2AX, suggesting that they do not introduce confounding genotoxic signals. Upon the addition of Etoposide, a significant increase in γ-H2AX fluorescence intensity was observed, demonstrating that the presence of these food matrices did not interfere with the detection of γ-H2AX (Figure 5).

**Figure 6:**
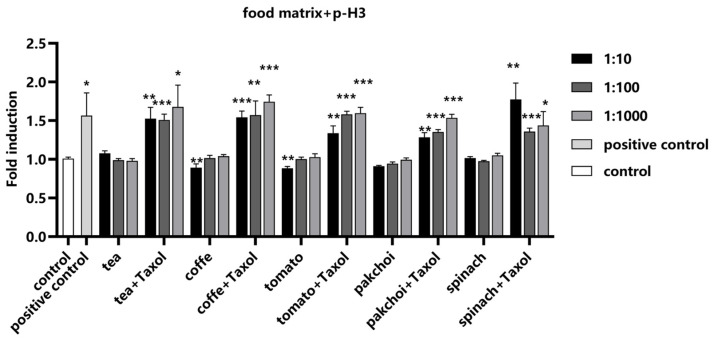
Impact of food matrices on the detection of p-H3. Food matrices, including coffee, tea, pak choi, spinach, and tomato, were serially diluted to 1:10, 1:100, and 1:1000 with serum-free medium. The diluted matrices were spiked with positive control Taxol (0.1 μM). The fluorescence intensity of p-H3 was measured and calculated as fold induction compared with control group. Statistical significance was determined using one-way ANOVA (* *p* < 0.05, ** *p* < 0.01, *** *p* < 0.001 vs. control).

Since 24 h Taxol treatment significantly increased the fluorescence intensity of p-H3 in Hep G2 cells, Taxol was used as a positive control to examine the effect of food matrices on the detection of p-H3. We found that the food matrices did not exert any influence on the detection of p-H3, demonstrating the robustness of the assay in the presence of potential matrix interference. The addition of Taxol resulted in a significant increase in fluorescence intensity, validating the effectiveness of the assay for the detection of aneuploid genotoxic compounds within the complex food matrices (Figure 6).

**Figure 7:**
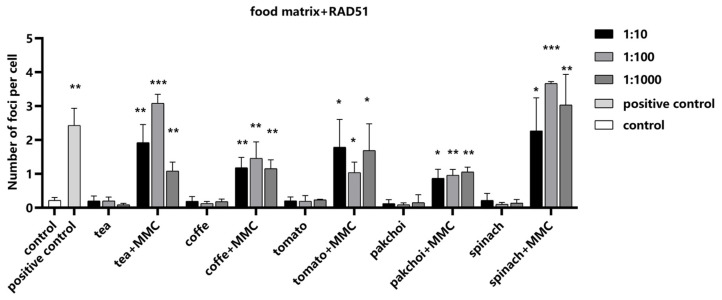
Impact of food matrices on the detection of RAD51. Food matrices, including coffee, tea, pak choi, spinach, and tomato, were serially diluted to 1:10, 1:100, and 1:1000 with serum-free medium. The diluted matrices were spiked with positive control MMC (10 μM). The average number of RAD51 foci in the nuclei per cell was measured. Statistical significance was determined using one-way ANOVA (* *p* < 0.05, ** *p* < 0.01, *** *p* < 0.001 vs. control).

Next, we investigated the influence of the food matrices on RAD51 detection, employing MMC as a positive control to ascertain the assay’s sensitivity to changes in RAD51 foci formation. Our findings indicated that the food matrices exerted no significant impact on the formation of RAD51 foci (Figure 7). Importantly, upon the addition of MMC, a pronounced increase in the number of RAD51 foci was observed, demonstrating that our assay can accurately detect RAD51 recruitment to DNA repair sites, even in complex food matrix backgrounds.

The above results underscore the superior throughput, time efficiency, and cost-effectiveness of our method compared with traditional in vitro assays for genotoxicity assessment, including the Ames assay, comet assay, micronucleus assay, and chromosome aberration assay. While our method shows promise, further validation with authentic contaminated food samples, such as food naturally contaminated with mycotoxins, is necessary to ensure the applicability and reliability of the method in real-world scenarios.

In summary, the multiparametric high-content analysis developed in this study holds promise as a robust tool for the detection and screening of genotoxic substances. By validating the assay’s performance in the presence of food matrices, we have taken a significant step toward implementing a reliable and sensitive screening method for genotoxic compounds in the food industry.

## 3. Materials and Methods

### 3.1. Reagents and Antibodies

RPMI-1640, DMEM, and fetal bovine serum (FBS) were purchased from Gibco (Grand Island, NY, USA). Anti-γ-H2AX mouse monoclonal antibody (80312) and Anti-p-H3 rabbit monoclonal antibody (3458) were obtained from Cell Signaling Technology (Danvers, MA, USA). Alexa-488-labeled anti-rabbit secondary antibody (A32731) and Alexa-488-labeled anti-mouse (A32723) secondary antibody were purchased from Invitrogen (Carlsbad, CA, USA). Fluorescent probe Hoechst 33342 was obtained from Life Technologies (Carlsbad, CA, USA). A panel of genotoxic agents was sourced from Shanghai Yuanye Bio-Technology Company (Shanghai, China), including Camptothecin, Etoposide, Chlorambucil, Cisplatin, Mitomycin C, 5-Fu, Taxol, Vincristine, Griseofulvin, Tozasertib, Azidothymidine and 4-NQO.

### 3.2. Cell Culture

Hep G2 and RAD51-EGFP-SW480 cells (Thermo Fisher Scientific, Rockford, IL, USA) were cultured in 1640 medium and high-glucose DMEM, respectively, and supplemented with 10% FBS at 37 °C with 5% CO_2_. Sixteen hours before the treatment, the cells were seeded into black-bottomed, 96-well plates at a density ranging from 1.5 × 10^4^ to 2 × 10^4^ cells per well. 

### 3.3. Treatment with Genotoxic Substances

The genotoxic substances used in this experiment are shown in Table 2. Genotoxic substances were introduced to cells at concentrations with cytotoxicity below 50% for a period of 24 h. Blank controls were established and maintained at 37 °C in a 5% CO_2_ incubator for 24 h. All chemical compounds were freshly prepared in dimethyl sulfoxide (DMSO) as stock solutions and serially diluted with serum-free medium immediately before the experiments. The diluted samples were added to each well (100 µL/well) at the indicated final concentrations.

### 3.4. Treatment of the Food Matrices

The vegetable samples of pak choi, spinach, and tomatoes were meticulously cleaned. They were then thoroughly chopped and blended to achieve a homogeneous mixture. The edible raw pulps were separated from the fibrous material by centrifugation at 12,000 rpm for 20 min at a temperature of 4 °C, yielding a clear and usable supernatant. The preparation of the tea matrix involved steeping 3 g of raw Pu-erh tea in 100 mL of boiling water for a duration of 15 min. Similarly, the coffee matrix was prepared by introducing 1 g of coffee powder to 80 mL of boiling water, followed by vigorous shaking to ensure complete dissolution. The resulting supernatants from both the tea and coffee infusions were carefully collected and subjected to filtration through a 0.22 μm filter to obtain a clear extract suitable for subsequent analysis. The above prepared matrices were diluted with serum-free medium at dilution ratios of 1:10, 1:100, and 1:1000 to serve as negative controls for assessing the genotoxicity of the food matrices. Etoposide (1 μM), MMC (0.1 μM), and Taxol (10 μM) were used as positive controls.

### 3.5. Immunofluorescence Assay

Cells were fixed with 4% paraformaldehyde (200 μL/well) for 15 min at room temperature. After fixation, the plates were washed twice with 100 μL/well phosphate-buffered saline (PBS). The cells were permeabilized for 15 min with 200 μL/well of permeabilization buffer (PBS containing 0.1% Triton X-100) at room temperature. After permeabilization, the cells were washed twice with 200 μL/well of PBS. The plates were blocked with 5% BSA (200 μL/well) for 1 h at room temperature to prevent nonspecific antibody binding. A total of 50 μL of primary antibody solution (containing 0.5% γ-H2AX or p-H3 primary antibody in 5% BSA) was added to each well, followed by a 2 h incubation at room temperature. After incubation, the primary antibody solution was aspirated, and the plates were washed twice with PBS. Then, 50 μL of secondary antibody solution containing Hoechst-33342 (0.2% secondary antibody and 0.01% Hoechst-33342 in PBS) was added to each well, and the plates were incubated for 1 h at room temperature in the dark. After this incubation, the wells were washed thrice with PBS. The wells were filled with 200 μL of PBS and sealed for scanning. Images were captured using a high-content cell analysis system (Opera Phenix, PerkinElmer, Waltham, MA, USA) and analyzed with Harmony 5.1 software (PerkinElmer, Waltham, MA, USA).

### 3.6. Statistical Analyses

All experiments were conducted with at least three independent replications. The intensities of γ-H2AX and p-H3, as well as the number of RAD51 foci for all compounds tested, were graphically represented using GraphPad Prism 8 software (GraphPad Software, Boston, MA, USA). Given that the sample means were normally distributed, one-way analysis of variance (ANOVA) was employed for comparisons, followed by Bonferroni post hoc correction. The statistical analyses were performed using GraphPad Prism 8 (* *p* < 0.05; ** *p* < 0.01; *** *p* < 0.001).

## Figures and Tables

**Table 1:** Summary of the effects of tested compounds on γ-H2AX, p-H3, and RAD51.

Compound Name	Minimum Effective Concentration (μmol/L)		
	γ-H2AX	p-H3	Rad51
AZT	1	10	10
4-NQO	0.1	1	1
5-Fu	1	1	10
Cisplatin	100	100	10
Camptothecin	0.1	1	1
MMC	0.1	0.1	0.1
Etoposide	1	10	10
Chlorambucil	10	10	1
Vincristine	1	1	0.01
Taxol	0.1	0.1	0.1
Griseofulvin	10	10	10
Tozasertib	10	0.1	0.1

Blue or yellow marks indicate a decrease or an increase in the parameter compared to the control group, respectively. Values in the table indicate the minimum effective concentration.

**Table 2:** Test compounds and summary of the results of in vitro genotoxicity assays.

Compound Name	CAS No.	Mode of Toxic Action	Ames	In Vitro Assays	DNA Strand Break	Reference
Camptothecin	7689-03-4	Topoisomerase I inhibitor	Pos	Pos	Pos	[25]
Etoposide	33419-42-0	Topoisomerase II inhibitor	Pos	Pos	Pos	[26]
Chlorambucil	305-03-3	DNA alkylation	Pos	Pos	Pos	[27]
Cisplatin	15663-27-1	cross-linking agent	Pos	Pos	Pos	[28]
Mitomycin C	1189805-46-6	cross-linking agent	Pos	Pos	Pos	[29]
5-Fu	51-21-8	Nucleoside analogue agents	Neg	Pos	Pos	[30]
Taxol	33069-62-4	Spindle poison	Neg	Pos	Neg	[3]
Vincristine	143-6709	Spindle poison	Pos	Pos	Neg	[3]
Griseofulvin	126-07-8	Spindle poison	Pos	Pos	Neg	[15]
Tozasertib	639089-54-6	Aurora A inhibitor	N/A	N/A	Neg	[3]
Azidothymidine	92586-35-1	Nucleoside analogue agents	Neg	Pos	Pos	[31]
4-NQO	56-57-5	DNA adduct agents	Pos	Pos	Pos	[1]

Pos, positive response; Neg, negative response; N/A, not available. Reported results of genotoxic tests of all 12 compounds are summarized. In vitro assays: combination of in vitro mouse lymphoma, chromosomal aberration, and micronucleus tests.

## Data Availability

Data are contained within the article. Further inquiries can be directed to the corresponding authors.
